# A Systematic Review of Evidence for a Role of Rest-Activity Rhythms in Dementia

**DOI:** 10.3389/fpsyt.2019.00778

**Published:** 2019-10-30

**Authors:** Stephen F. Smagula, Swathi Gujral, Chandler S. Capps, Robert T. Krafty

**Affiliations:** ^1^Department of Psychiatry, School of Medicine, University of Pittsburgh, Pittsburgh, PA, United States; ^2^Department of Epidemiology, Graduate School of Public Health, University of Pittsburgh, Pittsburgh, PA, United States; ^3^Veterans Administration, Veterans Integrated Service Network 4, Mental Illness Research, Education and Clinical Center of Excellence, Pittsburgh, PA, United States; ^4^Department of Biostatistics, Graduate School of Public Health, University of Pittsburgh, PA, United States

**Keywords:** rest-activity rhythms, sleep-wake rhythms, actigraphy, dementia, cerebrovascular disease, neurodegeneration

## Abstract

**Background:** Rest-activity rhythm (RAR) disruption may be a risk factor for dementia that can be objectively measured with wearable accelerometers. It is possible that risk monitoring and preventive interventions could be developed targeting RARs. To evaluate whether current evidence supports these applications, we systematically reviewed published studies linking RARs with dementia, its course, and mechanisms.

**Methods:** Entering pre-defined search terms in PsycINFO, MEDLINE, and PubMed databases returned 192 unique titles. We identified 32 articles that met our primary inclusion criteria, namely, that they examined objective RAR measures in the context of dementia, cognition, or brain biomarkers.

**Results:** Cross-sectional studies consistently found that people with dementia had less stable (5/6 studies), more fragmented (4/6 studies), lower amplitude rhythms (5/5 studies), that had a worse fit to 24-h models (3/3 studies). Findings from studies relating RARs to cognitive test performance (rather than diagnostic status) were more nuanced. RAR fragmentation was associated with neurodegeneration biomarkers in 2/2 studies; and 1/1 study found 24-h model fit related to hippocampal hyperactivation. Although 2/2 studies found RARs related to markers of cerebrovascular disease, the specific RARs and cerebrovascular disease measures were not consistent. Longitudinal studies (3/3 articles) reported that lower amplitude and worse 24-h rhythm fit predicted future cognitive impairment and executive function. However, interventions aimed at modifying RARs had mixed effects (e.g., 0/4 studies demonstrated effects of morning light on 24-h model fit; evening light was associated with improved 24-h fit in 2/2 studies reporting); these effects may be more evident in subgroups.

**Conclusions:** Consistent evidence shows that dementia is associated with disrupted RARs. Importantly, recent studies have shown that RAR disruption is associated with dementia biomarkers and, prospectively, with the risk of cognitive impairment. Interventions mostly tried using bright light to modify RARs in people who already have dementia; these studies produced modest effects on RARs and did not show modification of dementia’s course. Altogether, these findings suggest studies are needed to understand how RARs relate to changes in brain health earlier in the disease process. Better understanding of the biopsychosocial mechanisms linking RARs with future dementia risk can help further target intervention development.

## Introduction

**Figure 1:**
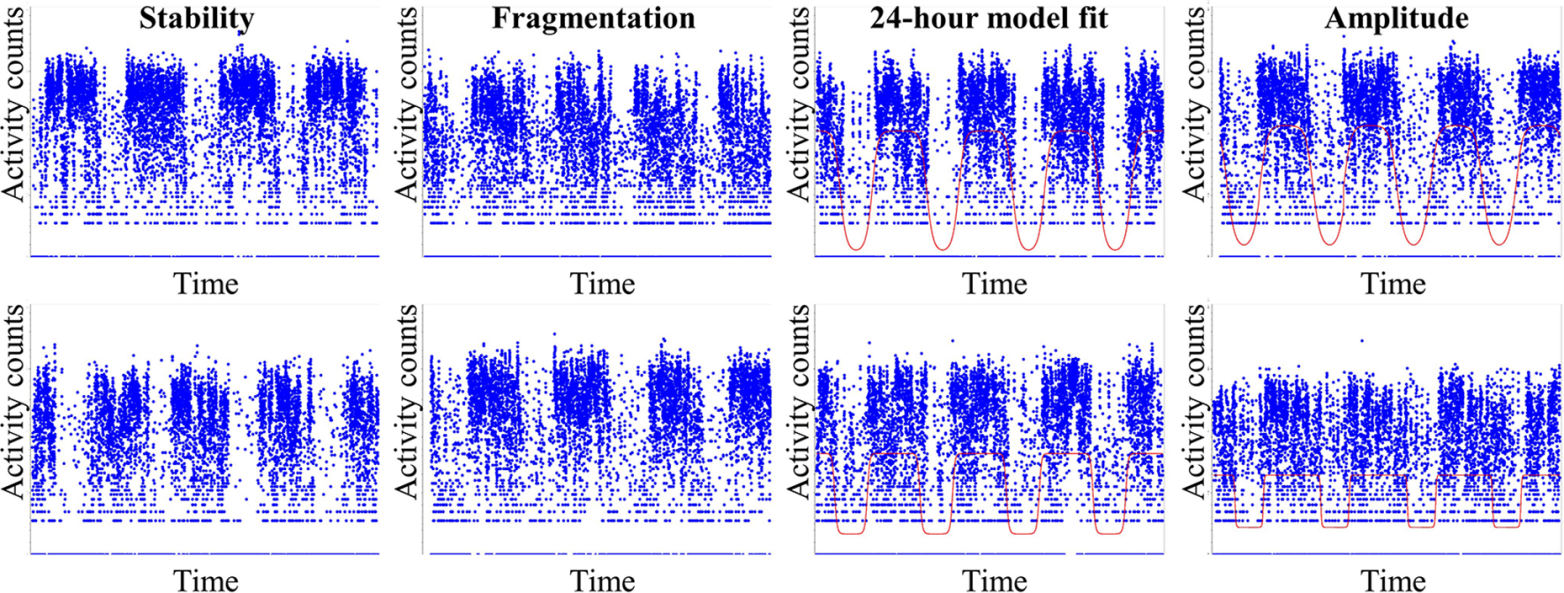
Commonly used rest-activity rhythm (RAR) measures. The top row represents high values on the given metric and the bottom row represents low values. From left to right: note stability, measured as nonparametric inter-daily stability, indicates how much the typical 24-h profile varies from day to day; fragmentation, measured as nonparametric intra-daily variability, indicates the frequency and extent of transitions in activity levels; 24-h model fit, from an extended cosine model (see line) pseudo-F statistic, indicates how well the data fits to a 24-h model; amplitude, derived from an extended cosine model, indicates rhythm height. More details on these common metrics are provided in the reviewed articles and elsewhere, e.g., see the study by Smagula (2).

The first case report documenting rest-activity rhythm (RAR) disruption in people with dementia was published in 1986 (1). This confirmed observations of clinicians and caregivers but had the advantage of objectively specifying the activity patterns that characterized people with dementia. RAR characteristics including regularity, fragmentation, amplitude, and fit to 24-h models can be objectively quantified by applying a variety of methods to several days of continuous accelerometer recordings (see brief description of RAR methods in **Figure 1**).

**Figure 2:**
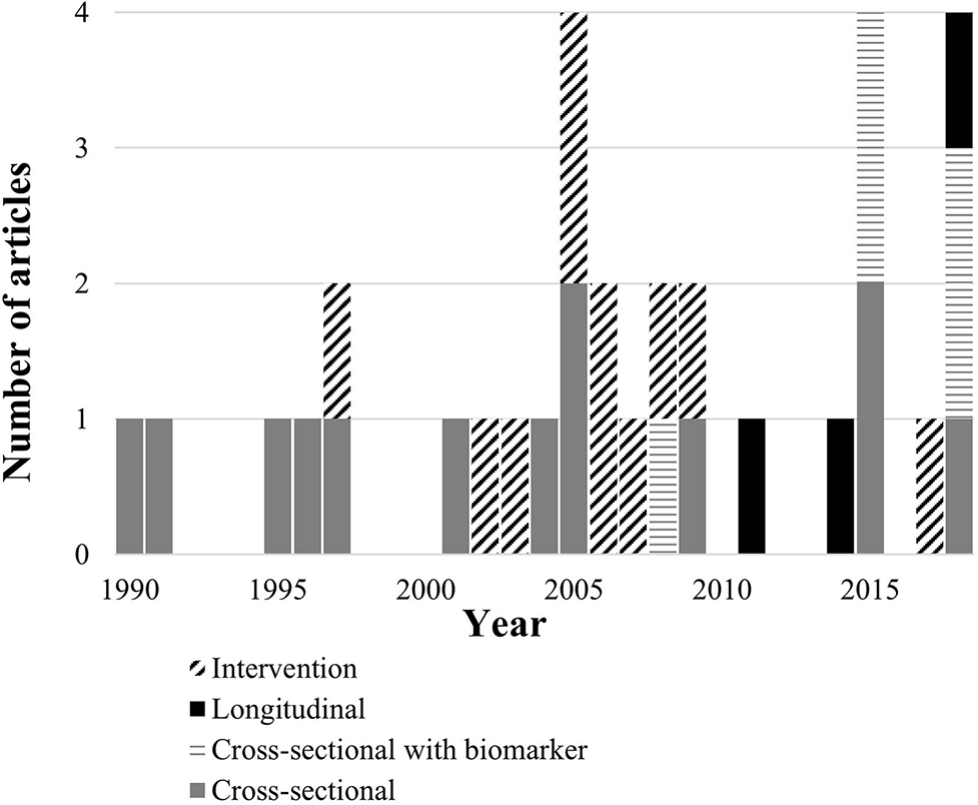
Number of articles identified by year and study design.

Almost three decades later, literature on relationships of RARs with dementia and cognition continues to grow. Published research currently includes observational, biomarker, and intervention studies (**Figure 2**). Now that consumer-friendly accelerometer technology is increasingly available, clear evidence for a role of RARs in dementia’s course could lead to useful new clinical applications. Specifically, risk stratification approaches could be designed to detect RAR characteristics that mark or hasten dementia risk. Preventive interventions could also be developed targeting RARs to protect brain health and cognition.

But, the likelihood that these clinical applications will succeed, and what specifically they should target, depends on knowing 1) what aspects of RARs affect the course of cognitive aging and its underlying mechanisms, 2) whether these aspects of RARs are modifiable, and 3) if doing so improves cognitive aging. To identify gaps between current knowledge and potential clinical applications of wearable technology targeting RARs, we systematically reviewed evidence relating RARs, brain health, and cognition.

## Methods

*Target article inclusion criteria:* We included articles that reported results from data-based human subject research studies with more than 20 participants (i.e., we excluded review articles, animal studies, small pilot studies, and case reports). In addition, studies were included only if they examined cognition, a biomarker related to cognition in aging, Alzheimer disease, or a related dementia (except for Parkinson’s due to differences in the biological mechanisms and low number of Parkinson’s articles). To be included, articles were required to have quantified 24-h RAR variable(s) from objectively measured activity time series data. We only included articles that were written in English.

**Figure 3:**
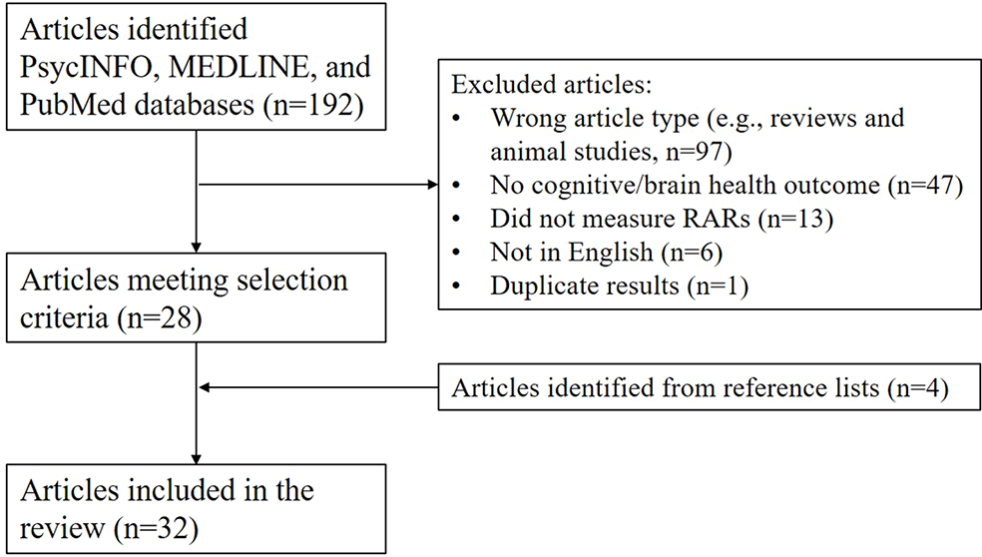
Search process and article selection.

*Article search and selection process* (**Figure 3**): We searched PsycINFO, MEDLINE, and PubMed databases using pre-specified terms [(“sleep-wake rhythm” or “rest-activity rhythm” or “activity rhythm”)) AND (“Alzheimer’s” or “dementia” or “cognition” or “cognitive” or “neurodegenerative” or “cerebrovascular”)]. To minimize bias and errors in article selection, two raters (SFS and CC) independently reviewed the title, abstract, and text (as needed) of the 192 returned articles to determine if they met article inclusion criteria. Rating discrepancies were minimal and resolved *via* consensus discussion referencing the article’s text. Returned articles were excluded because 97 were not the correct article type, 47 did not have a cognitive or relevant biomarker outcome, 13 did not examine objectively quantified RARs, 6 were not in English, and 1 reported the same results in two studies. Next, to minimize bias and errors, two raters (SFS and SG) reviewed each article and recorded selected summary information (see **Supplemental Tables**). Finally, the frequency and consistency of statistical results were then summarized across design, exposures, and outcome factors.

## Results

The search process outlined above returned 28 articles, meeting our inclusion criteria. Four additional articles were identified from their reference lists. Most studies used cross-sectional observational designs (n = 18) and five of these included biomarkers. Intervention studies were also common (n = 11). Few studies used longitudinal observational designs (n = 3).

*Cross-sectional studies(***Table 1***):* Consistent evidence demonstrated that people with dementia had less stable rhythms (5/6 studies 3–8). For example, the recent work by Saito et al. (5) demonstrated that, among nursing home residents, those with dementia had lower IS values (reflecting less stability across days). The one study (8) that failed to detect an overall statistically significant overall association between dementia and RAR stability did find stability was lower among people with dementia who were living in institutional settings (compared with community dwelling people with dementia and healthy controls); the sample of people with Alzheimer’s disease living independently in this study was small (n = 8).

**Table 1:** Summary of evidence relating RAR and cognitive measures.

		Studies comparing RARs by cognitive status	Study examining associations of RARs with cognitive test performance
Cross-sectional studies	Stability (IS)	5/6 studies reported associations (3–7). The one nonsignificant study (8) was mixed: people with dementia had lower IS (compared with controls) if living in a nursing home but not community settings	1/2 studies: Luik et al. (9) found lower IS related to worse performance on measures of processing speed and executive function, whereas Oosterman (10) did not.
	Fragmentation (IV)	4/6 studies reported associations (3, 4, 7, 11). Note that Harper et al. (4) found frontal-temporal dementia, but not AD, was associated with higher IV. Van Someren et al. (8) and Saito et al. (5) failed to find associations between IV and cognitive status.	2/2 studies: Luik et al. (9) and Oosterman et al. (10) found associations of higher IV with slower processing speed and worse executive function.
	Goodness of fit measures (R^2^, pseudo-F, or 24-h autocorrelation coefficient)	3/3 studies reported associations (4, 6, 12) but results differed by disease. Aharon-Peretz et al. (12) found patients with multi-infarct dementia had worse fit than AD and controls. Harper et al. (4) found frontal-temporal dementia had worse fit than AD and control.	Gehrman et al. (13) did not find an association between overall rhythm robustness with global dementia severity. However, among participants with less robust RARs, less severe dementia was associated with higher F statistic, steeper, and wider RARs. Among those with more robust RARs, less severe dementia was associated with earlier acrophase and narrower active periods.
	Amplitude (cosine-based model or nonparametric)	5/5 studies reported associations (4, 6, 7, 14, 15)	
Longitudinal studies	Goodness of fit measures (pseudo-F)	2/2 (16, 17) studies found having lower rhythm robustness and lower amplitude was associated with future cognitive status. Tranah et al. (16) found women with lower robustness and amplitude were more likely to have dementia or MCI approximately 5 years later. Rogers-Soeder et al. (17) found men with lower robustness and amplitude were more likely to have clinically significant cognitive decline approximately 3 years later.	Walsh et al. (18) reported that lower robustness was associated executive function task performance approximately 5 years later; these associations were attenuated after adjustment for other health factors.
	Amplitude (cosine-based model)		Walsh et al. (18) reported that lower amplitude was associated with executive function task performance approximately 5 years later (independent of adjustments for other health factors).

In addition, 4/6 studies (positive studies (3, 4, 7, 9 and nonsignificant studies 5, 8) found that cognitive impairment was related to higher RAR fragmentation. For example, the early work by Wittin et al. (3) demonstrated that Alzheimer disease patients had higher intra-daily variability, reflecting more fragmented (less consistent) rhythms. All of the studies examining goodness-of-fit and amplitude measures found that cognitive impairment was associated with less robust rhythm fits and lower amplitude rhythms (3/3 4, 6, 12 and 5/5 4, 6, 7, 14, 15, respectively).

Findings regarding associations of RARs and cognitive performance have been more nuanced. Both Luik et al. (9) and Oosterman et al. (10) showed that RAR fragmentation related to processing speed and executive function measures; furthermore, Luik et al. (9) found that the association of fragmentation and executive function was stronger among older compared with younger participants. Luik et al. (9) also found stability related to these cognitive measures, but Oosterman et al. (10) did not. Gehrman et al. (13) did not find an association between rhythm model fit and global dementia severity.

*Biomarker studies (***Table 2***):* Only five studies investigated RARs and dementia biomarkers. Three studies assessed markers of neurodegenerative processes (21–23: 1) Musiek et al. (22) found fragmentation related to markers of amyloid deposition; 2) Van Someren (21) reported that greater fragmentation correlated with lower medial temporal lobe volume better than any other risk factor studied; and 3) Sherman et al. (23) noted a relationship between 24-h rhythm fit and memory performance that was statistically mediated by anterior hippocampal hyperactivation during an associative memory task.

**Table 2:** Summary of evidence relating RAR and neurobiological measures.

Stability (IS)	Lower IS was associated with: • Cerebral microbleeds (p = 0.06) (19) • Occiputal periventricular and frontal white matter lesions in Oosterman et al. (20); not global white matter lesions in Zuurbier et al. (19) • Lower medial temporal lobe volume, but this association was attenuated when adjusting for age (21).
Fragmentation (IV)	Higher IV was associated with: • Greater levels of amyloid deposition markers in the brain and cerebrospinal fluid (22). • Lower medial temporal lobe volume (21) • White matter lesion volumes in Zuurbier et al. (19) but not Oosterman et al. (20)
Goodness of fit measures (pseudo-F)	Better fit was associated with better memory performance, and this association was statistically mediated by hyperactivation in the hippocampus (23)
Amplitude (cosine-based model or nonparametric)	Lower amplitude was associated with lower medial temporal lobe volume, but this association was attenuated when adjusting for age (21).

Two studies linked RAR measures with cerebrovascular disease markers. Oosterman et al. (20) found that white matter lesions were related to RAR stability, but Zuurbier et al. (19) did not; however, note that, while Oosterman et al. found associations in occipital periventricular and frontal regions, but not elsewhere, Zuurbier et al. examined only whole brain white matter lesion volume and not regional levels. Zuurbier et al. (19) found an association of RAR fragmentation with overall white matter lesion burden, and while Oosterman et al. did not (20), correlations of RAR fragmentation with white matter lesion volumes were similar in both studies (r = 0.10–0.12 in the study by Oosterman et al. (20) and 0.10 in the study by Zuurbier et al. (19); sample sizes differed substantially: 135 (20) and 970 (19).

*Longitudinal studies (***Table 1***):* In both studies reporting (16, 17), lower rhythm robustness (24-h model goodness-of-fit measure) and amplitude were associated with future cognitive decline. In terms of changes in cognitive test performance, Walsh et al. (18) found that lower robustness and amplitude were associated with executive function test performance 5 years later; in this study, the association of amplitude, but not robustness, was independent of other health characteristics (e.g., BMI, medical morbidities, medication use, and physical activity levels). Note that these prospective studies all included over 1,000 participants.

*Intervention studies (***Table 3***):* An early report noted that unattended daytime bright light has beneficial effects on RAR stability and fragmentation among patients with intact vision (31). However, additional studies of bright light interventions have shown modest or no effects on rhythms in people with dementia. Regarding the strength of intervention evidence, note (**Supplemental Table 4**) that published studies almost always focused on pre-post effects (rather than whether pre-post effects differed by randomized group) and always had follow-up periods of less than 3 months; therefore, these interventions were all considered studies with “level II” evidence strength according to previously published criteria (30). Two studies (27, 28) did report controlled results. That said, the intervention studies have shown:

**Table 3:** Summary of light interventions targeting RARs.

Intervention	Effects on:			
	Stability (IS)	Fragmentation (IV)	Goodness of fit	Amplitude
Morning bright light	No intervention effects in the study by Dowling et al. (24), although stability increased in patients who had aberrant rhythms (defined as their least active 5 h beginning after 3 AM).	Dowling et al. (24) found no intervention effects.	0/4 studies observed intervention effects (24–27). Ancoli-Israel (26) observed a trend for improvement in the pseudo-F statistic in the morning light group (p = 0.06).	0/3 studies (24–26) although Dowling et al. (24) found that relative amplitude increased in people with aberrant rhythms at baseline.
Morning bright light + Melatonin	No effects (28)		Positive effects light+melatonin (27) on cosine-based amplitude	
Afternoon			No effect (26)	No effect (26)
Evening bright light			2/2 studies showed significant pre-post improvements (28, 29)	0/2 studies showed effects (28, 29)
Daytime (unattended) bright light	Improvements but only in patients with intact vision (30)			

An early report noted that unattended daytime bright light had beneficial.

- Morning bright light did not have statistically significant effects on RAR stability (25), fragmentation (25), 24-h model fit (24–27)| and amplitude (24–26). Among people classified as having aberrant rhythms at baseline, however, Dowling et al. (25) observed that morning light was associated with increases in stability and a trend towards increased amplitude. One controlled study (28) found that morning bright light plus melatonin has positive effects on 24-h model fit and amplitude.
- Afternoon light had no effects on 24-h model fit and amplitude in one study reporting controlled results (27).
- Evening light did have significant pre-to-post intervention effects on 24-h model fit in the two studies reporting (24, 26), no effects on amplitude were observed.

A few reports examined effects of other intervention approaches. Two studies (29, 32) using peripheral electrical stimulation did not have effects on stability or fragmentation. Moving to a small-scale care facility (33) and a hand movement intervention (34) also did not have statistically significant effects on RARs. Finally, one study found that 2 weeks of institutional care lead to a dampening (relative amplitude reduction) of RARs that recovered after returning home (35).

## Discussion

In summary, this systematic review of available evidence found consistent evidence that RAR disruption is more common in people with dementia. New evidence from large studies shows that RAR disruption predicts future cognitive decline. Evidence for these associations is strong, given the consistency of results across studies and the wide range of sample sizes and populations examined. Recently, biomarker studies have shown RAR disruption relates to the neurobiological hallmarks of dementia; however, as discussed below, these studies have been in relatively small cross-sectional samples and therefore cannot infer temporality in these relationships. Finally, note that several studies investigated whether light interventions modify RAR disruption or the disease course in people who already have dementia. These studies did not consistently show light interventions modify RARs; the most promising findings were from two studies using evening light (24, 26), one study using a combination of morning light and melatonin (28), and when analyses restricted to patient subgroups (those with intact vision (23 and RAR disruption at baseline 25).

The largest and most consistent evidence indicates that RAR disruption is more common in people who have dementia. Every study identified found that people with dementia have lower activity levels (reflected in RAR amplitude measures) (4, 6, 7, 14, 15), and most studies found that people with dementia have more fragmented (3, 4, 7, 11) less stable (3–8) rhythms, that fit a 24-h pattern relatively poorly (4, 6, 12). While these past studies were limited by their cross-sectional design, evidence from recent large prospective cohort studies have shown that RARs disruption also predicts future cognitive function (in people free of dementia at study baseline) (16, 17). However, it is not yet clear whether RAR disruption relates to dementia and dementia risk as a pathogenic contributor or as a downstream marker of existing pathology.

When reaching the severity that typically mandates institutional care (the setting where most RAR research began), people with dementia have already experienced significant neurodegeneration and likely have cerebrovascular disease (36). This macrostructural damage can affect the neural circuits that control circadian rhythms including the suprachiasmatic nucleus (37). Thus, it is not surprising that people with dementia frequently exhibit RAR disruption. Advanced-stage pathology could also explain why, in cases of overt dementia, increasing exogenous signals to the circadian system *via* light produces mixed or no effects (e.g., light may be inputting on already-damaged circadian circuits that have little remaining control effects).

Furthermore, recent studies have shown that RAR characteristics are associated with the neurobiological biological hallmarks of dementia early in the disease process (i.e., in community and pre-clinical samples) including amyloid deposition (22), medial temporal lobe atrophy (21), and cerebrovascular disease (although associations may be region specific) (19, 20). In addition, the study by Sherman et al. showed that RAR disruption related to hippocampal hyperactivation (23), which is considered a component of pre-clinical dementia pathophysiology related to early memory decline (e.g., see the study by Smagula et al. (38). In this context, findings from longitudinal epidemiological studies (showing RAR disruption predicts future cognitive decline) might be due to RARs already reflecting early-stage neuropathology (which was not controlled for in existing longitudinal studies). Thus, available evidence does not resolve whether RAR disruption hastens the progression of, or simply reflects, early-stage pathology to dementia.

However, there are other sources of evidence that suggest rhythm disruption can contribute to dementia etiology; for example, an animal model of circadian misalignment affects neuronal structure (39), and chronic jet-lag (disrupting natural rhythms) is associated with reduced temporal lobe volume (40). Future prospective observational studies can be designed to determine whether RARs reflect or affect the pathogenesis of dementia. If RAR fragmentation in everyday human life uniquely contributes to the progression of amyloid pathology and neurodegeneration, then interventions aimed at modifying RAR fragmentation would be warranted. Alternatively, if RARs do not contribute to the progression of dementia above and beyond pre-existing neurobiological pathology, then interventions modifying RARs are less likely to succeed (although measuring RARs might still be useful for risk stratification).

Thus, longitudinal and intervention studies are needed to place associations of RARs and brain health in greater context. Apparent discrepancies in available literature may be because the role of RAR differs by age, life stage, or pre-existing pathology (which vary across study samples). It is not clear why associations between RAR fragmentation and cognition are stronger in older subsets of the sample in the study of Luik et al. (9) The meaning or role of RAR stability might differ depending on life stage (e.g., pre- or post- retirement) or other factors like the presence of cardiovascular risk factors, sedentary behavior, or poor sleep. Differences in these factors across samples could explain why Luik et al. (community sample; sample average age = 59) (9) found stability related to cognitive test performance, but Oosterman et al. (clinic-based sample; average age = 69) (10) did not. Future longitudinal studies are needed to identify the modifiable factors that lead to RAR disruption.

Psychosocial and physical activity have been associated with stronger RARs in people with dementia (41). This suggests that boosting or maintaining activity participation would be a logical approach to modify RARs. But, there remains a need to clarify which, of several inter-related risk factors (RARs, physical activity, social activities, and sleep), have the most potent and unique affects on brain health, and at what point in life. Our review of existing evidence suggests that interventions to curtail effects of RARs on brain health may require targeting specific subgroups (25, 31) and/or using multiple approaches (28).

## Conclusion

Decades of research has produced solid evidence that people with dementia are more likely to exhibit RAR disruption. Recent studies have demonstrated that, even in pre-clinical samples, RARs relate to early markers of dementia neurobiology and may increase dementia risk. Several questions remain to be answered. Different measures of RAR robustness appear related to dementia neurobiology and risk, but future research is needed to identify the specific aspects of RARs that best signal these processes. Furthermore, it remains unclear how relationships between RARs and brain health play out time, what leads to RAR disruption, and whether modifying RARs at different stages in disease pathogenesis could prevent dementia. Available evidence supports the idea of promoting healthy RARs to prevent dementia, but doing so may require intervening early in disease pathogenesis and using multiple approaches (e.g., scheduling activity, bright light, and sleep interventions).

## Data Availability Statement

All datasets generated for this study are included in the article/**Supplementary Material**.

## Author Contributions

SS and RK conceptualized the project and literature search. SS, CC, and SG performed the search and tabulated the results. SS drafted the manuscript, and all authors critically revised the manuscript.

## Funding

This work was supported by K01MH112683 (to SS) and R01GM113242 (to RK). SG is supported by a VA VISN 4 MIRECC Competitive Pilot Grant and a Veterans Research Foundation Gerald Goldstein Early Career Mental Health Research Award. The authors also acknowledge Annemarie Luik, PhD, for providing helpful feedback on the content of this manuscript.

## Conflict of Interest

The authors declare that the research was conducted in the absence of any commercial or financial relationships that could be construed as a potential conflict of interest.
