# Supplementary material for: A Systematic Review of Evidence for a Role of Rest-Activity Rhythms in Dementia
Source: Front Psychiatry. 2019 Oct 30;10:778. doi: 10.3389/fpsyt.2019.00778 (PMC6832024; doi:10.3389/fpsyt.2019.00778)
Supplement: Supplementary file 1 [file DataSheet_1.docx]

**Table S1. Cross-Sectional Studies (N=13) of RARs and Dementia**

**Table S2. Longitudinal Studies (N=3) of RARs and Dementia**

**Table S3. Biomarker Studies of RARs and Dementia (N=5)**

**Table S4. Intervention Studies (N=11) of RARs and Dementia.**
